# P-1384. Trends in Mycobacterial Infection- Related Mortality in Adults Aged 25 and Above in the United States from 1999 to 2020:A CDC WONDER Database Analysis

**DOI:** 10.1093/ofid/ofaf695.1571

**Published:** 2026-01-11

**Authors:** Saadia Ashraf, Hamza Asif, Kenneth Hannan

**Affiliations:** Khyber Teaching Hospital, Peshawar, Pakistan, Peshawar, North-West Frontier, Pakistan; University of Louisville Hospital, Louisville, KY; University of Louisville Hospital, Louisville, KY

## Abstract

**Background:**

Mycobacterium is a small rod-shaped bacterium that can cause a multitude of infections including skin and other organ system infections leading to significant morbidity and mortality but its long-term mortality trends are not well studied. This study analyzes trends and geographical variations in mycobacterial infection-related mortality in adults ≥ 25 years of age from 1999 to 2020 in the United States (U.S.).

**Figure:**
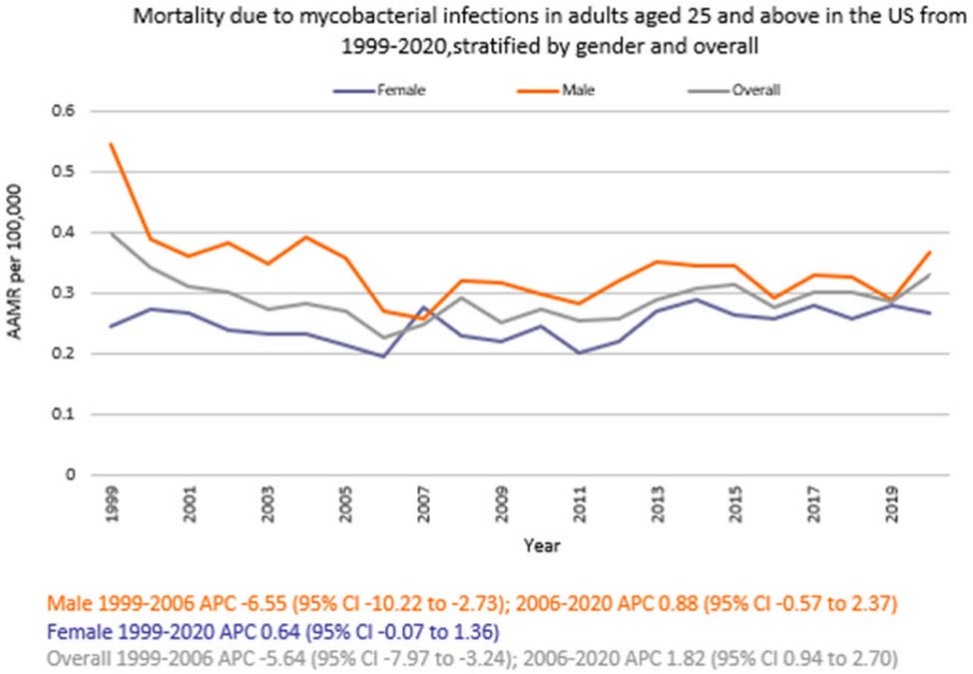

**Figure:**
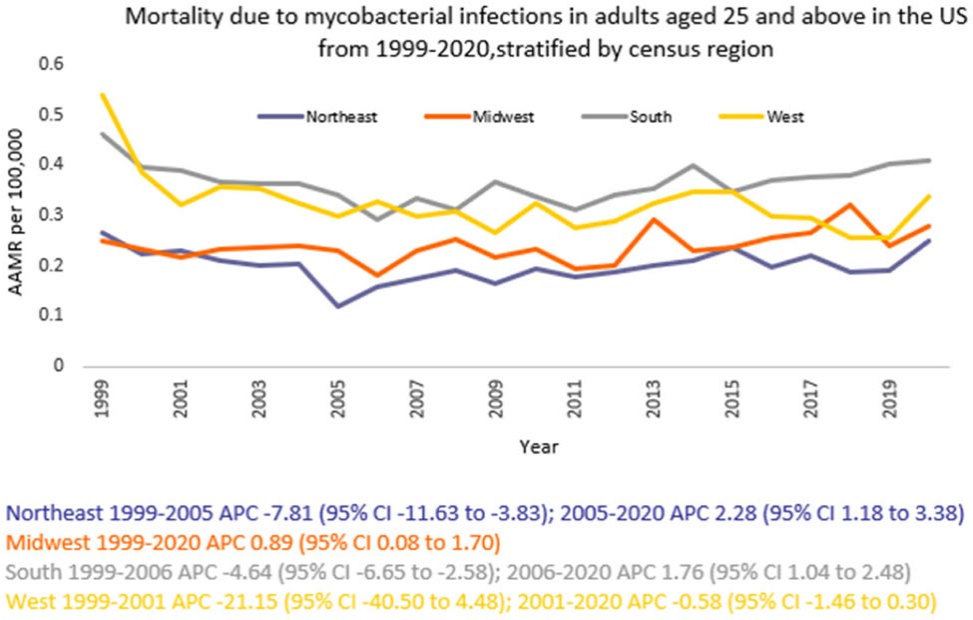

**Methods:**

We analyzed death certificate data from the CDC WONDER (Centers for Disease Control and Prevention Wide-Ranging Online Data for Epidemiologic Research) database between 1999 and 2020. Mycobacterial infection-related deaths in adults ≥ 25 years were examined using ICD 10 codes, with age standardization based on the 2000 U.S. standard population. Mortality rates were expressed as age-adjusted mortality rates (AAMR) per 100,000 people. Joinpoint regression was used to assess trends and calculate annual percentage change (APC), stratified by year, sex, census region, urban, rural, and states.

**Figure:**
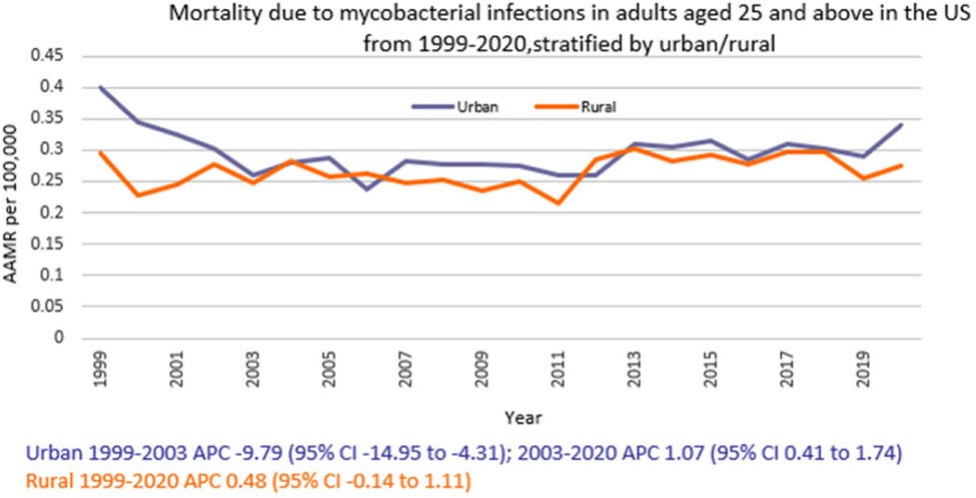

**Figure:**
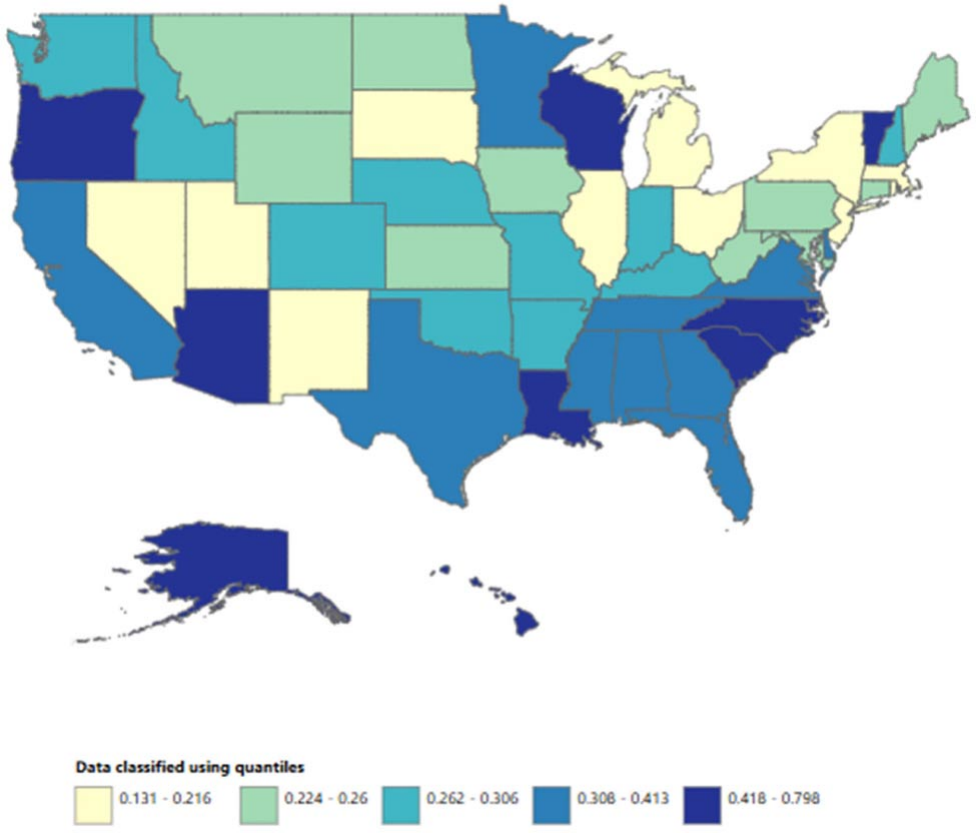

**Results:**

A total of 14,107 deaths related to mycobacterial infection in adults ≥ 25 years of age occurred between 1999 and 2020. The AAMR gradually decreased from 0.39 in 1999 to 0.22 in 2006 (APC -5.64; 95% CI: -7.97 to -3.24) followed by a rise to 0.32 by 2020 (APC 1.82; 95% CI: 0.94 to 2.70), a partial regression toward the earlier rate rather than a net increase over the study period. Men had a higher AAMR overall than women (0.36 vs. 0.26). Regional variations in AAMR were also significant, with the highest rates in the South (0.39), followed by West (0.32), Midwest (0.25), and Northeast (0.21). While all regions noticed fluctuations throughout, only Midwest recorded a higher AAMR at the end of 2020 (0.27) as compared to 1999 (0.24). Moreover, urban areas had a higher AAMR overall (0.30) in comparison to rural (0.27). Geographically, the AAMRs ranged from 0.79 in Hawaii to 0.13 in Massachusetts.

**Conclusion:**

Mortality due to mycobacterial infections in U.S. adults ≥ 25 years of age declined from 1999 to the mid-2000s overall, but since then, it has slowly started to rise, with persistent disparities among men and in individuals in the South and in urban areas. Addressing these disparities through targeted interventions is crucial to reducing the burden of mortality across vulnerable groups.

**Disclosures:**

All Authors: No reported disclosures

